# Computational Pathology with Topological signatures and Visual Word Encoding

**DOI:** 10.21203/rs.3.rs-7724033/v1

**Published:** 2025-12-01

**Authors:** Taymaz Akan, Richa Aishwarya, Md. Shenuarin Bhuiyan, Mohammad Alfrad Nobel Bhuiyan

**Affiliations:** LSU Health Shreveport; LSU Health Shreveport; LSU Health Shreveport; LSU Health Shreveport

**Keywords:** Computational pathology, Topological data analysis, Persistent homology, image classification, Machin Learning

## Abstract

Tissue analysis is considered the gold standard for the diagnosis of a wide spectrum of disorders. However, pathologists perform labor-intensive evaluations to ensure accurate results. Computational pathology has made significant advances in the development of task-specific predictive models. Nevertheless, traditional pixel- or texture-based features often fail to capture both local and global structural patterns together with their spatial organization. This study developed TopoBoW, a computational framework to objectively characterize morphological patterns using local and global microscopy image features. We developed TopoBoW by integrating Topological Data Analysis (TDA) and Bag-of-Visual-Words (BoVW), combining it with an attention-guided multi-layer perceptron (MLP) trained to distinguish between healthy and pathological muscle tissue. TDA captures global structural features with Betti curves derived from persistent homology, whereas BoVW encodes local textural patterns with SURF descriptors and histogram encoding. We also utilized visualizations to examine the statistical behavior of feature vectors across disease classes and healthy controls, evaluating their discriminative ability. We compare TopoBoW with several baseline and advanced models, including TDA, histogram of oriented gradients (HOG) with XGBoost and Attention-based MLP. TopoBoW demonstrated state-of-the-art performance on muscle tissue classification and outperformed all baselines in terms of all classification criteria, including accuracy, F1-score, and AUC. With its interpretable feature-based computational framework, TopoBoW can assist with pathological research, education, and interactive diagnostic workflows by integrating global structural and local textural information from images.

## 1 Introduction

Tissue pathology reflects a wide range of structural and cellular changes resulting from physiological conditions, aging, injury, and disease. These changes often result in altered tissue architecture, cell morphology, nuclear positioning, and extracellular matrix organization [1]. Such morphological changes are significant biomarkers for the diagnosis, monitoring of disease progression, and assessment of therapeutic efficacy in a wide range of medical conditions, including genetic, neurological, and metabolic diseases. For instance, diseases like diabetes and amyotrophic lateral sclerosis (ALS) frequently cause pathological alterations in skeletal muscle, including fiber atrophy, nuclear mislocalization, and altered extracellular matrix composition [2], [3]. Histological analysis remains the gold standard for detecting such tissue abnormalities, but traditional methods rely heavily on manual annotations by trained experts. Even more so in large-scale or longitudinal studies, this labor-intensive and time-consuming manual process is affected by inter- and intra-observer variability [4], [5]. Consequently, there is a growing demand for objective, interpretable, and scalable computational tools to assist in pathology analysis across a variety of tissue types and disease contexts.

Computational pathology has undergone significant changes in recent years due to the rise of digital slide scanning, AI research advancements, increased access to large datasets, and high-performance computing resources [6], [7], [8]. This field has huge potential for automating clinical diagnosis, predicting patient prognosis and response to therapy, and discovering new morphological biomarkers using tissue imaging.

The clinical practice of pathology involves a wide range of tasks, including metastasis detection [9], [10], subtyping [11], [12], grading [13], [14], and staging. Given the huge variety of possible disease presentations, pathologists are often required to address highly diverse diagnostic challenges simultaneously [8], [15], [16]. In order to meet this complexity, computational pathology has successfully employed a variety of methods, such as deep learning, handcrafted feature extraction, and hybrid approaches, each tailored to specific tasks [17], [18], [19], [20], [21]. However, despite these promising developments, many existing approaches fall short in capturing the full spectrum of tissue morphology, particularly when both global structural patterns and local textural details must be jointly analyzed for reliable and interpretable outcomes.

Despite these advancements, it is still challenging to extract clinically significant features from WGA-stained images. Conventional techniques like ImageJ-based tools are time-consuming and produce varying outcomes due to their manual cell counting, segmentation, and thresholding. Moreover, the majority of existing automated pipelines use classical image processing or CNN-based segmentation, which are frequently limited to fiber boundary detection or average size quantification [22], [23], [24], [25], [26], [27]. These approaches may overlook higher-order structural properties, such as fiber spatial arrangement, global tissue organization, or disease-related shape irregularities.

Furthermore, the majority of developed automated methods have been primarily developed and validated on H&E-stained images [28], [29], [30], [31], where muscle fiber boundaries are less distinct and are frequently simplified using basic geometric assumptions, such as circular or elliptical area estimations (i.e., approximating each muscle fiber as a perfect circle or ellipse to compute cross-sectional area). On the other hand, WGA-stained images demonstrate a precise delineation of the sarcolemma, which reveals the original irregularity and complexity of muscle fiber contours. These detailed boundaries present a challenge in terms of the application of traditional shape-based metrics, such as cross-sectional area or diameter, without a substantial reduction in structural detail. Consequently, to accurately interpret the morphological complexity captured in WGA-stained muscle sections, more sophisticated and robust analysis techniques are necessary, particularly in diseased tissues where the shape of the fibers is frequently highly variable.

In this context, topological data analysis (TDA) offers a promising alternative. TDA can extract global shape features that are invariant to local deformations and noise by characterization the connectivity and voids within tissue architecture. Betti curves are a frequently employed TDA tool that summarizes the evolution of topological features (e.g., connected components, holes) across filtration thresholds, thereby providing an interpretable descriptor of tissue geometry [32].

In addition to TDA, local texture and color data within muscle images can be efficiently captured using the Bag of Visual Words (BoVW) technique, which is derived from natural language processing [33]. BoVW models visual content by extracting local descriptors (e.g., SURF [34]), clustering them into a visual vocabulary, and encoding images as histograms over this vocabulary. This representation preserves fine-grained patterns of color, shape, and texture, which may correspond to pathological microstructures in diseased muscle.

In this study, we propose a hybrid feature extraction framework that fuses TDA-derived Betti curve features with BoVW histograms to create a unified representation of pathological image morphology. We apply this approach to WGA-stained sections of five different skeletal muscles—quadriceps, gastrocnemius, soleus, extensor digitorum longus (EDL), and tibialis anterior (TA)—collected from healthy wild-type mice and two disease models: G93A*SOD1 mice, a well-established model for ALS, and C57BL/6-Ins2Akita/J (Akita) mice, a Type I diabetes model. The pathological characteristics identified in these models, including central nucleation, variability in fiber size, and structural disarray, are amenable to assessment through both topological and texture-based descriptors.

This study aims to advance pathological image analysis by integrating global structural summaries (TDA) with local appearance cues (BoVW). This integration improves the capacity to discriminate between healthy and diseased muscle tissues. The fused features improve classification performance while also providing interpretable insights into disease morphological signatures, paving the way for scalable and objective skeletal muscle phenotyping in preclinical research.

## 2 Materials and methods

### 2.1 Animal Models and Tissue Preparation

Skeletal muscle samples were collected from two established mouse models of disease: G93ASOD1 transgenic mice, which are a model of amyotrophic lateral sclerosis (ALS), and C57BL/6-Ins2Akita/J (Akita) mice, which are a model for Type I diabetes. Healthy controls were established using wild-type and G93ASOD1 non-carrier mice on a C57BL/6 background. Both the disease models and their respective control mice were acquired from The Jackson Laboratories (Bar Harbour, ME, USA). All mice were housed in standard conditions, which included a typical chow diet, unlimited access to water, 12-hour light-dark cycles, and temperature-controlled environments. All animals were purchased from The Jackson Laboratory (Bar Harbor, ME, USA), a commercial research animal supplier, and were not privately owned. The LSU Health-Shreveport Institutional Animal Care and Use Committee approved the animal care and experimental procedures in accordance with the NIH Guide for the Care and Use of Laboratory Animals. The specifics of muscle dissection and sample processing have been previously described [35].

### 2.2 WGA Staining and Microscopy

Skeletal muscle tissues were obtained from five distinct anatomical regions: the quadriceps, gastrocnemius, soleus, extensor digitorum longus (EDL), and tibialis anterior (TA). All mice were anesthetized with isoflurane (Isospire^™^, Dechra Veterinary Products, KS, USA) overdose (5% concentration), and anesthesia was confirmed by the absence of a pedal withdrawal reflex before euthanasia by exsanguination and pneumothorax. Tissues were, then, extracted bilaterally, fixed in formalin, and paraffin-embedded to prepare histological blocks. Deparaffinization and rehydration were performed on thin sections (5 μm). Antigen retrieval was accomplished by boiling the samples in 10 mmol/L sodium citrate buffer (pH 6.0) at 100°C. In order to label cell membranes, sections were incubated with Alexa Fluor 488-conjugated wheat germ agglutinin (WGA, 5 μg/mL) for 1 hour after being blocked in a solution containing 1% bovine serum albumin, 0.1% cold water fish skin gelatin, and 1% Tween-20 in PBS. The nuclei were counterstained with DAPI for a period of 5 minutes. The slides were washed in PBS and mounted using Vectashield mounting medium. The images were analyzed using Nikon NIS Elements (v4.13.04), and the Nikon A1R confocal microscope (20× objective) was used to conduct the imaging. The complete staining and imaging protocol has been comprehensively described in previous research [35], [36].

### 2.3 Dataset and Classification Framework

The final dataset comprised five distinct muscle types per subject, including WGA-stained muscle sections from Akita, G93A*SOD1, and wild-type mice. Centrally located nuclei, an increase in small or irregularly shaped fibers, and altered myofiber morphology are some of the pathological changes observed in G93A*SOD1 mice, a model that is representative of ALS [35], [37], [38]. Conversely, healthy muscle fibers are distinguished by their variable cross-sectional area (CSA), peripheral nuclei, and polygonal geometry [11]. Histological abnormalities and muscle dysfunction are the distinguishing characteristics of the Akita mouse, which functions as a model for Type I diabetes [39], [40]. For the purpose of model development and evaluation, all images were classified as either healthy or diseased (e.g., diabetic muscle or ALS). In order to evaluate the classification framework’s performance, the dataset was partitioned into training and test sets.

### 2.4 Computational Feature Extraction and Classification

We propose a hybrid classification framework for histology image analysis that combines classical computer vision features with topological descriptors. We apply this approach to automatically classify skeletal muscle histology images into healthy, ALS, or diabetic categories. To guarantee consistency across the dataset, all input images were resized to a fixed resolution of 224 × 224 pixels before feature extraction.

First, we employed the BoVW model to capture textural and morphological information. For each image in the training set, Speeded-Up Robust Features (SURF) were extracted to identify key local interest points. These features were then clustered using K-Means to create a unified “visual vocabulary.” Subsequently, each image in the dataset was represented as a histogram corresponding to the frequency of these visual words, effectively summarizing the textural content of the image. Next, TDA features were implemented to characterize the structural organization of the muscle fibers. These pre-computed TDA features offer a distinctive, global perspective on tissue architecture by quantifying high-level spatial relationships, including the cyclic structures and connectivity patterns formed by the muscle cells. Finally, the TDA feature vector and BoVW histogram of each image were combined to create a single, all-inclusive hybrid feature vector. Local textural patterns are combined with global topological signatures in this fused vector. The fused feature vectors from the training set were used to train an attention-based multilayer perceptron (MLP).

#### 2.4.1 Bag-of-Visual-Words (BoVW) Using SURF Descriptors

We implemented a BoVW model to extract local visual patterns from WGA-stained images. This model pipeline was constructed as follows:

##### I. Keypoint Detection and Description:

Speeded-Up Robust Features (SURF) were extracted for each image. Each image I was represented by a set of local descriptors $D=\{d_{1},d_{2},\ldots ,d_{N}\}$, where $d_{i}\in R^{k}$.

##### II. Visual Vocabulary Construction:

All descriptors from training images were clustered into K clusters using MiniBatch K-Means. The centroid of each cluster represented a “visual word,” forming the vocabulary $V=\{v_{1},v_{2},\ldots ,v_{K}\}$.

1 $$\underset{V}{\text{min}}\sum_{i=1}^{N}\underset{j=1,\ldots ,K}{\text{min}}{\left\|d_{i}-v_{j}\right\|}^{2}$$

For this study, we set K=150.

##### III. Histogram Representation:

Each image was encoded as a histogram $h\in R^{K}$, where each element $h_{j}$ denotes the frequency of visual word $v_{j}$ in the image. We refer to this histogram representation as the BoVW feature vector for an image.

#### 2.4.2 TDA Features

To capture the higher-order structural and spatial information of muscle fibers, we incorporated topological features derived from persistent homology.

##### I. Persistence Diagrams:

Using Cubical Persistence, we computed 0D and 1D persistence diagrams ${PD}_{0}$ and ${PD}_{1}$ from RGB channel (Red, Green, Blue) alongside grayscale image [41].

Let $f:Z^{2}\to R$ express the pixel intensity function. The sublevel sets of f define a filtration, and persistent homology tracks the birth and death of connected components $\left(H_{0}\right)$ and loops $\left(H_{1}\right)$.

##### II. Betti Curve Representation:

Each persistence diagram was converted to a Betti curve $\beta_{k}(t)$ using 100 bins for each channel, where k∈{0,1}.

The Betti curve counts the number of topological features alive at threshold *t*.

#### 2.4.3 Feature Concatenation:

The Betti curves from grayscale, red, green, and blue channels were concatenated, resulting in a final 800-dimensional vector per image:

2 $$x_{\text{TDA}}=\left[\beta_{0}^{\text{gray}\backslash :},\beta_{1}^{\text{gray}\backslash :},\beta_{0}^{\text{red}\backslash :},\beta_{1}^{\text{red}\backslash :},\ldots \right]\in {\mathbb{R}}^{800}$$

**Figure 1:**
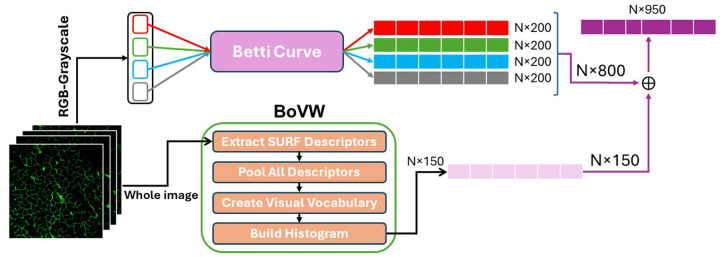
Overview of the hybrid feature extraction pipeline. Topological features are obtained using Betti curves from persistence barcodes across multiple color channels, while visual features are extracted using the BoVW approach based on SURF descriptors. The resulting topological and visual histograms are concatenated to form the final feature vector for classification.

By concatenating the vectors obtained from the TDA and BoVW pipelines, we implemented a feature-level fusion strategy to take advantage of complementary information captured by topological and visual descriptors. Figure 1 shows how the TDA features were calculated using Betti curves for each topological channel (Red-TDA, Green-TDA, Blue-TDA, and Gray-TDA). This made a fixed-size representation that shows multi-scale topological summaries across color channels. We also computed BoVW histograms by taking SURF descriptors from input images, using k-means clustering to construct a visual vocabulary, and then encoding each image as a normalized histogram over visual word occurrences. The final feature representation for each image was obtained by concatenating the TDA-based topological feature vector with the BoVW histogram. This fused vector integrates global structural signatures (from persistent homology) and local texture-color statistics (from visual words), enhancing the model’s capacity to distinguish disease phenotypes in skeletal muscle morphology. All features were standardized prior to classification.

#### 2.4.4 Classification

To harness both local (BoVW) and global (TDA) representations, we concatenated the normalized feature vectors from both methods for each sample per $x_{\text{hybrid}}=[x_{\text{TDA}},x_{\text{BoVW}}]\in R^{950}$, where 800 dimensions are from TDA and 150 from BoVW. The concatenation of BoVW and TDA features produced the fused feature vectors, resulting in a 950-dimensional feature vector per sample (800 from TDA and 150 from BoVW). The 200-dimensional representations that were taken from each RGB channel and grayscale (i.e., 200 × 4 = 800) made up the TDA features. Each sample was then truncated to 950 features in order to guarantee dimensional consistency across all input vectors. For the classification task, all samples were numerically encoded in binary form and labeled as either disease or non-disease.

Prior to classification, the dataset was partitioned using stratified 20-fold cross-validation, which preserved the class balance in each training and validation split. This approach ensured that all samples contributed to both model training and evaluation, while preventing data leakage and maintaining robust performance estimation. All processing steps were applied consistently across folds with fixed random seed initialization to enable reproducibility.

##### Model Architecture

Our classification architecture is a neural network designed to process 950-dimensional feature vectors using a Multi-Head Attention mechanism. The input vector is initially molded into a series of eight segments with 475 dimensions each. This sequence is processed by a 10-head Multi-Head Attention layer with 0.1 dropout, which learns the contextual inter-dependencies between the feature segments. To improve generalization, we apply feature-level noise augmentation during training. Specifically, Gaussian noise with a standard deviation of 0.05 is added to the 950-dimensional input vector before it is reshaped for attention processing.

**Figure 2:**
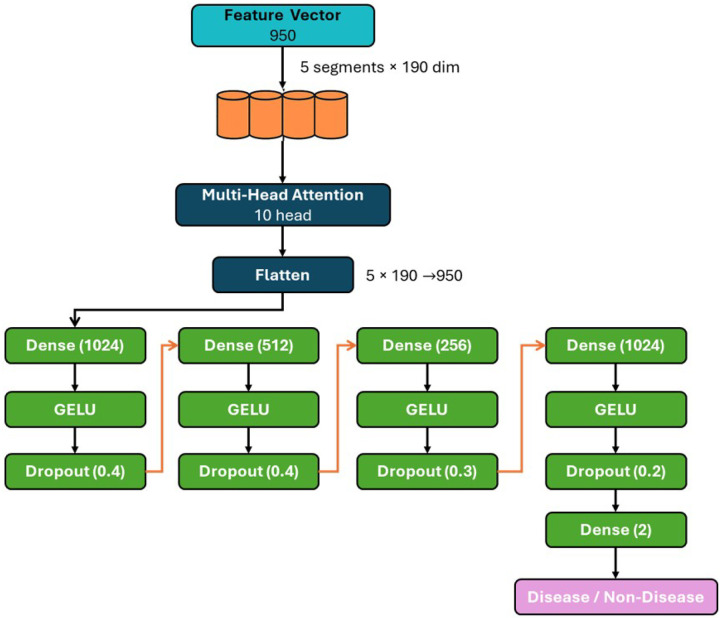
Architecture of the classification model. The 950-dimensional hybrid feature vector is first passed through a multi-head attention mechanism (10 heads), followed by flattening. The output is processed through a series of fully connected layers with GELU activation and dropout regularization. The final dense layer with two output units performs binary classification to distinguish between disease and non-disease samples.

This augmentation is only applied during training and has no effect at inference time. The resulting sequence is flattened and passed into a deep Multi-Layer Perceptron (MLP) for classification. This classifier head is composed of four sequential blocks: a dense layer (1024 units) with GELU activation and 0.4 dropout; a dense layer (512 units) with GELU activation and 0.4 dropout; a dense layer (256 units) with GELU activation and 0.3 dropout; and a final dense layer (1024 units) with GELU activation and 0.2 dropout. This deep structure creates a feature bottleneck that encourages the model to learn a compressed yet rich representation. The network concludes with a 2-unit dense output layer for the final binary classification. The architecture of the proposed classifier is illustrated in Fig. 2. It successfully distinguishes between diseased and non-diseased cases by processing the features through a multi-head attention mechanism and a series of dense layers.

## 3 Results

Histological image analysis facilitates the identification of structural changes associated with numerous diseases. This study examines skeletal muscle tissue by analyzing WGA-stained sections to evaluate myofiber size alterations and categorize samples into ALS, diabetic, and healthy groups across various muscles, including the quadriceps, gastrocnemius, tibialis anterior, extensor digitorum longus, and soleus.

We created the TopoBoW model to automate the classification process, enabling it to learn distinguishing features from patterns in histopathology. To evaluate the efficacy of our proposed TopoBoW model for classifying skeletal muscle pathology, extensive comparisons against several baseline and advanced models, including TDA, histogram of oriented gradients (HOG) with XGBoost and Attention-based MLP. WGA-stained histopathological images of diverse muscle types were employed in the classification task to distinguish between ALS, diabetic, and healthy skeletal muscle tissue.

### 3.1 Configuration

We conducted hyperparameter adjustment for all methods with XGBoost classifiers to attain optimal model performance. Cross-validation was employed to meticulously refine critical parameters, including the learning rate, maximal tree depth, number of estimators, and subsampling ratio. The optimum configurations were determined based on validation accuracy. The HOG + XGBoost model was specifically constructed with 300 estimators, a learning rate of 0.01, a maximum depth of 7, and a subsample ratio of 0.6. The TDA + XGBoost model achieved its highest accuracy with 200 estimators, a learning rate of 0.05, a maximum depth of 6, and a subsample ratio of 0.8. The XGBoost was implemented in the TopoBoW + XGBoost model with 100 estimators, a default learning rate of 0.3, and a maximum depth of 6. For MLP, the AdamW optimizer was employed to optimize the model for 500 epochs, with a learning rate of 10e − 4 and learning rate scheduling via cosine annealing. The final evaluation was conducted using the model that demonstrated the highest level of performance for each fold, as designated by the validation accuracy.

### 3.2 Quantitative Evaluation

Quantitative evaluation criteria, including precision, recall, F1-score, accuracy (with standard deviation), and area under the curve (AUC), are given in Table 1. In this table, WGA-stained skeletal muscle images are used to compare the classification performance of various feature-based models to the proposed TopoBoW technique. The TopoBoW model combined with attention achieved the highest performance across all evaluation metrics, with a precision of 0.97, recall of 0.98, F1-score of 0.97, accuracy of 0.98, and an AUC of 0.98. In contrast, the TopoBoW + XGBoost model had a remarkable performance, achieving an F1-score of 0.87 and an AUC of 0.94, however it did not surpass the attention-based alternative. Traditional models such as HOG + XGBoost and HOG + Attention showed notably lower performance, particularly in recall and F1-score, indicating limited capacity to capture discriminative morphological features. TDA-based models outperformed their HOG-based counterparts, highlighting the stronger discriminative capacity of topological features. Specifically, the TDA + Attention model achieved an AUC of 0.90 and an F1-score of 0.83. The TopoBoW + Attention model demonstrated the most successful performance in all metrics, demonstrating the efficacy of integrating topological and visual representations within an attention-guided learning framework.

**Figure 3:**
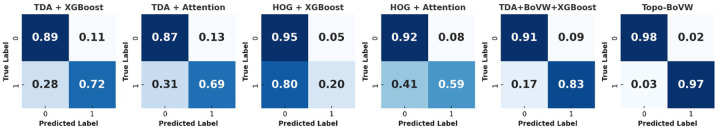
Confusion matrices demonstrate the classification performance of six different models on WGA-stained skeletal muscle images. With its superior sensibility and accuracy, the TopoBoW model effectively distinguishes between healthy and diseased muscle tissue, surpassing other models in terms of false positives and true negatives.

Figure 3 shows the confusion matrices for six classification models based on various topological and visual features. Models using only TDA or HOG features, such as TDA + XGBoost or HOG + XGBoost, performed unevenly, particularly in classifying diseased tissue (class 1). Moreover, the performance of both TDA and HOG was slightly enhanced by the inclusion of attention mechanisms, which suggests that local context refinement may be valuable for feature discrimination. It is worth noting that the integration of global structural alongside local visual features led to a significant improvement when TDA and BoVW were combined (TDA + BoVW + XGBoost). Finaly, the Topo-BoVW model outperformed the others, with a classification accuracy of 98% across every category. Integrating topological signatures with BoVW encoding effectively captures morphological and textural features that are critical for disease classification.

**Figure 4:**
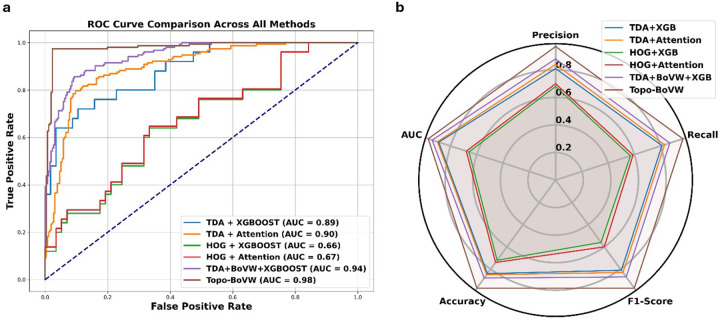
**(a)** Compare all models’ classification performance with ROC curves. The TopoBoW model had the highest AUC of 0.98, indicating excellent discrimination. HOG-based models had lower AUC values and limited classification capacity. **(b)** Radar plot showing precision, recall, F1-score, accuracy, and AUC for each method. The TopoBoW model consistently outperformed all other approaches across all metrics in capturing topological and visual features for skeletal muscle pathology classification.

Figure 4 illustrates a detailed comparison of model performance. The TopoBoW model demonstrated superior discrimination abilities with an AUC of 0.98, as shown in the ROC curves (Fig. 4a). Alternative TDA-based architectures, such as TDA + BoVW + XGBoost and TDA + Attention, exhibited commendable performance, achieving AUC values of 0.94 and 0.90, respectively. The HOG-based models, on the other hand, had much lower AUC values and a severely limited ability to classify data. The radar plot (Fig. 4b) represents a comprehensive view of performance across five key metrics. TopoBoW consistently outperformed, with the highest values for precision, recall, F1-score, accuracy, and AUC. These findings support the benefit of combining topological and visual features with attention-based learning for robust muscle disease classification.

### 3.3 Exploratory Feature Analysis

We conducted a series of visualizations intended to describe the statistical behavior of feature vectors across the two classes: disease (diabetic or ALS) and non-disease (healthy control) in order to evaluate the discriminative ability of the extracted features, which included BoVW and TDA representations. These plots aid in determining which feature types contribute to class separability and help confirm whether feature distributions differ significantly across classes.

By visualizing raw values, class-wise averages, variability, and projections, we aimed to

- Determine the global patterns of class separation across the 950 features.,
- Determine which feature blocks (e.g., Gray-TDA, RGB-TDA, BoVW) differ the most across conditions,
- Determine whether the learned features meaningfully encode disease-specific muscle morphology.

**Figure 5:**
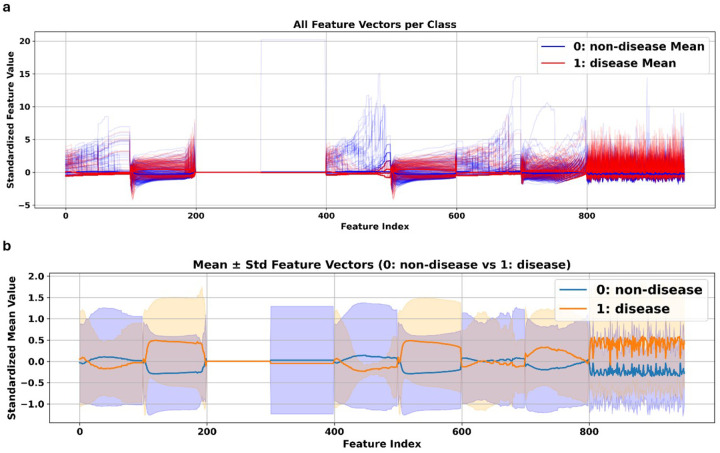
**(a)** Standardized feature vectors for all samples plotted per class. Each line represents the 950-dimensional feature vector of a single image, with semi-transparent curves showing individual samples and bold lines denoting class-wise means (blue: non-disease, red: disease). Regions of pronounced divergence suggest features with high class-separability. **(b)** Class-wise mean and standard deviation (mean ± std) for each feature dimension. Shaded regions represent one standard deviation around the mean value for each class.

Figure 5a shows all of the standardized feature vectors from the dataset, with each class’s vectors overlay on top of each other. Each line indicates the full 950-dimensional feature vector of one sample. Class 0 (non-disease) is shown in blue, and Class 1 (disease) is shown in red. The bolded curves are associated with the class-wise means, while the semi-transparent lines reflect individual sample variations. This visualization provides a quick overview of how features behave across classes and highlights regions of concentrated variance and divergence. In particular, features in the BoVW region (Last 150 features) along with certain color-specific TDA blocks, especially the Green and Blue channels, show a considerable separation between classes, highlighting their higher discriminatory potential. In contrast, features derived from the red channel (indices 200–400) show minimal divergence, likely due to the negligible signal in this channel, as seen in the WGA-stained image in Fig. 1. Representative WGA-stained skeletal muscle image, in which green fluorescence highlights extracellular matrix boundaries. The absence of red signal corresponds with the low relevance of red-channel-derived features observed in Fig. 1.

This analysis is further illustrated in Fig. 5b, which shows the class-wise mean and standard deviation for each feature dimension. Shaded bands indicate one standard deviation around the mean, and solid lines represent the standardized mean feature values in the plot. Each feature group (Gray-TDA, Red-TDA, Green-TDA, Blue-TDA, and BoVW) is visually segmented to aid interpretability. This figure demonstrates that Class 1 (disease group) has a more consistent morphological phenotype, with less variability in TDA-derived features in particular. In contrast, the non-disease class (Class 0) displays higher variability, especially in the Gray-TDA and Red-TDA groups. Importantly, the BoVW features again show marked separation between the two classes.

**Figure 6:**
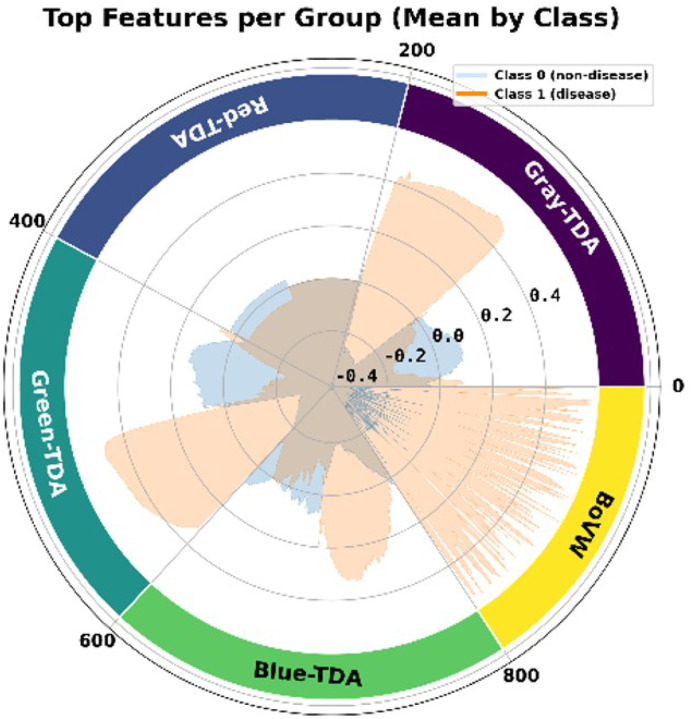
Radar plot illustrating the class-wise mean values of standardized features, which are categorized according to their origin: Gray-TDA, Red-TDA, Green-TDA, Blue-TDA, and BoVW. Each region represents a different set of features, with Class 0 (non-disease) in light blue and Class 1 (disease) in orange.

The radar plot of the mean feature values for each class is shown in Fig. 6 for a comprehensive, angular class-wise view of the feature behavior. The 950 features are arranged circularly, and colored regions denote class-wise magnitude differences across angular segments corresponding to feature groups. This plot demonstrates that the BoVW, Gray-TDA, and Green-TDA regions present the most pronounced class separations, highlighting their predominant role in the differentiation of diseased from non-diseased tissue. Although Blue-TDA features exhibit certain class-wise variation, the separation is less consistent. Conversely, the Red-TDA features demonstrate a significant degree of overlap between classes, which suggests that they have limited discriminative value and is indicative of the minimal red signal present in the original WGA-stained images.

**Figure 7:**
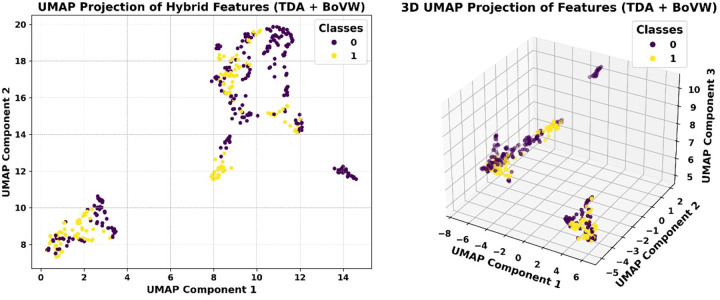
UMAP visualizations of hybrid feature vectors with both topological (TDA) and visual (BoVW) representations. **(a)** 2D UMAP projection with class labels (0: no disease, 1: disease) that shows different clustering patterns between the two groups. **(b)** 3D UMAP projection further highlights the class-wise separability in the embedded space, supporting the discriminative capacity of the hybrid feature set.

Figure 7a and 7b show two- and three-dimensional UMAP projections to determine whether the extracted hybrid features preserve meaningful structure in a reduced latent space. These plots demonstrate that the features capture class-relevant patterns even after nonlinear dimensionality reduction, as evidenced by the minimal overlap between clusters of disease and non-disease samples. Despite the reduction in nonlinear dimensionality, these plots show clearly defined clusters of disease and non-disease samples with little overlap. The 2D embedding (Fig. 7a) clearly demonstrates bifurcated groupings, whereas the 3D projection (Fig. 7b) confirms the stability of class separation in multiple views and provides additional spatial depth. These representations prove that the hybrid feature structure is effective at encoding morphological features that are specific to diseases.

**Figure 8:**
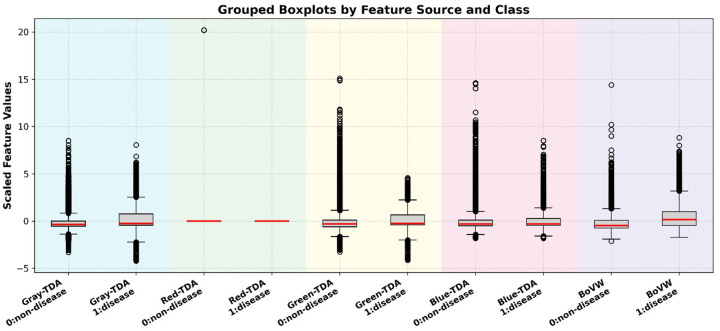
Grouped boxplots show scaled feature values from different feature sources (Gray-TDA, Red-TDA, Green-TDA, Blue-TDA, and BoVW) and classes (0: no disease, 1: disease). Each pair of boxplots represents the distribution of all features extracted from a given source, separated by class. Red-TDA features show little variation, but other groups, in particular BoVW, Blue-TDA, and Gray-TDA, show clear differences in median and spread between classes, reflecting their relative discriminative capacity.

Finally, Fig. 8 presents grouped boxplots of standardized feature values across both classes, segmented by feature source. The 950-dimensional feature vector was split into five blocks: Gray-TDA, Red-TDA, Green-TDA, Blue-TDA, and BoVW. Each pair of class-specific boxplots is positioned side by side and overlaid on a soft background to visually separate feature groups. This plot enables comparative assessment of intra-group and inter-class variation. It further confirms that BoVW features and Blue-TDA exhibit the largest differences between classes, while Gray-TDA and Green-TDA are largely overlapping. This group representation provides a brief but useful summary of how each feature category contributes with the classification challenge.

BoVW features appeared as the most discriminative in terms of both statistical variation and latent space structure, always showing clearer class-wise separation across visualizations. Among the TDA-derived features, those extracted from the Blue channel exhibited the greatest discriminative capacity, while features from the Gray and Green channels showed moderate separation. In contrast, Red-channel features demonstrated minimal variance and limited utility in distinguishing between classes. It is worth mentioning that Class 1 disease samples showed less variation across different feature groups. This could reflect the presence of consistent morphological abnormalities such as atrophied fibers and centrally located nuclei, as highlighted by WGA staining. Collectively, these observations verify that the combined feature representation, which incorporates BoVW and multi-channel TDA, effectively encodes class-relevant structural information and facilitates its application for precise disease classification.

**Figure 9:**
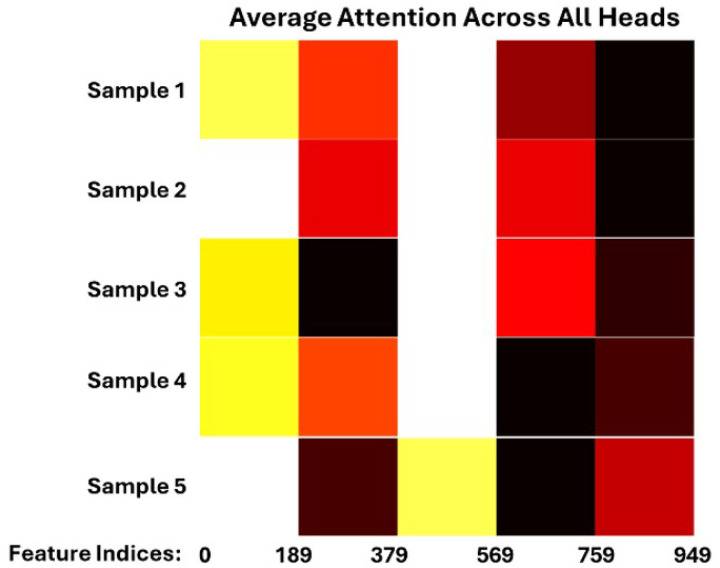
Attention heatmap over the 950-dimensional feature space, visualized for 5 randomly selected test samples. The model divides the input feature vector into five equal-length segments (190 features each) for attention processing.

Although the BoVW-derived features appear discriminative—particularly in the region spanning the last 150 indices of the 950-dimensional feature space—the corresponding attention map indicates relatively low attention weights in that segment. The attention mechanism divides the full feature vector into five equal segments (each with 190 features), and BoVW features occupy the final segment (indices 760–949), with only 150 out of 190 positions used. The classification model may be prioritizing global structural information captured by topological features over localized visual patterns, as illustrated by the observed reduction in attention within this region (See Fig. 9 that visualized for 5 randomly selected test samples). This implies that, for differentiating between ALS and diabetic conditions in WGA-stained skeletal muscle images, the model relies more heavily on the global organization and spatial topology of muscle fibers than on fine-grained texture or shape descriptors encoded by BoVW.

## 4 Discussion

In this study, we developed an automated pathological image analysis pipeline termed TopoBoW to evaluate skeletal muscle pathology in two disease models: ALS and diabetic myopathy. The parallel comparison with TopoBoW and manual approach showed that the TopoBoW pipeline can deliver relatively accurate measurements of muscle features. This is expected to facilitate the evaluation of therapeutic impact and the pathophysiological studies of muscle diseases.

The most critical steps involve the identification of muscle fiber boundaries in WGA-stained sections, which allows for the computation of fiber cross-sectional areas (CSA), evaluation of fiber size variability, and detection of shape abnormalities. These structural features, along with the spatial correlation between muscle fibers, assist in the diagnosis of conditions such as ALS and diabetic myopathy, which necessitates expert knowledge and intensive manual labor. On the other hand, the proposed model can find disease-related patterns directly from raw image features, which means it doesn’t need to do explicit CSA calculations. This could lead to uncovering hidden pathological patterns that are not readily captured through manual analysis.

Our model circumvents these limitations by integrating topological signatures to capture global morphological structure and BoVW to encode local texture and color patterns. The model leverages complementary representations of muscle tissue without requiring explicit CSA measurement or manual annotation. The incorporation of an attention mechanism facilitates better classification performance by means of adaptive focus on the most discriminative features across these heterogeneous domains. Together, this design facilitates the identification of disease-specific tissue alterations in a more effective and interpretable manner.

Our exploratory feature analysis provides further insight into the model’s decision-making process. BoVW features demonstrated the highest-class separability, possibly due to their adaptability to local anomalies in fiber texture and intensity patterns. TDA-derived features, except for the red channel’s features, showed substantial discriminatory power, reflecting the prominence of WGA fluorescence in these spectral bands. In contrast, the red-channel features exhibited minimal variance, which is consistent with the weak red signal in the original images. However, the attention heatmaps of the model demonstrated that the BoVW segment of the feature vector receives relatively low attention, despite its class-discriminative potential. This implies that the model might prioritize global features over local patterns during classification.

Furthermore, disease-class samples have less variability across most feature groups, indicating a more homogeneous pathological tissue morphology. This is in line with the recognized histological characteristics of ALS and diabetic myopathy, which include increased central nucleation, fiber atrophy, and loss of structural integrity. These features are more consistent in affected samples than in healthy muscle tissue, which exhibits greater heterogeneity.

Despite our promising findings, our study has a number of limitations. First, the dataset was constructed from a limited animal models, each representing a specific disease state (ALS or diabetes) and genetic background. While this design enables controlled comparisons, it may restrict the generalizability of the model to other types of myopathies or to human tissue. Secondly, the WGA staining protocol is capable of achieving exceptional membrane delineation; however, it fails to capture intracellular features, including fibrosis, inflammation, or mitochondrial alterations, which are also pertinent to the progression of the disease. Third, the classification task was framed as a binary distinction between healthy and diseased tissue, thereby oversimplifying the heterogeneity that exists within and across disease types. Furthermore, although the BoVW and TDA features are interpretable to a certain extent, they are still representations that are either handcrafted or engineered. The model does not currently incorporate spatial context or anatomical muscle-specific priors, which may be important in more complex classification settings. Finally, although the attention-based classifier improves interpretability and performance, it remains a black-box model to some extent, and future work should explore methods such as feature attribution techniques to improve transparency and trustworthiness in biomedical applications.

## 5 Conclusions

This study proposes TopoBoW, an interpretable and efficient framework for automated skeletal muscle pathology classification. Thanks to integrated BoVW and TDA features, the model captures not only local texture but also global tissue structure, which leads to a more accurate classification of ALS, diabetic, and healthy muscle tissue. Although BoVW features showed the highest statistical separability, attention-based analysis revealed that the model assigned lower attention to this segment. This suggests that disease classification in WGA-stained muscle sections may rely more on global architectural organization than on local texture irregularities. Moreover, the reduced variability observed in diseased samples supports the presence of consistent morphological signatures associated with pathology. This study has certain limitations, despite the promising outcomes. These include a relatively small dataset, limited disease diversity, and the lack of spatial context in feature representation. In order to enhance interpretability and clinical relevance, future research will expand the dataset, apply feature attribution techniques, and incorporate anatomical priors.

## Figures and Tables

**Table 1:** Comparison of classification performance across traditional feature-based models and TopoBoW on WGA-stained skeletal muscle images. Metrics reported include precision, recall, F1-score, accuracy (with standard deviation where applicable), and AUC. The proposed fusion with attention-based model outperformed all baselines in all evaluation criteria using 20-fold cross-validation.

Method	Train/Test	Precision	Recall	F1-Score	Accuracy	AUC
TDA + XGB [42]	20-fold	0.81	0.81	0.81	0.841 (± 0.041)	0.89
TDA + Attention	20-fold	0.84	0.83	0.83	0.854% (± 0.081)	0.90
HOG + XGBOOST	20-fold	0.68	0.57	0.56	0.724 (± 0.049)	0.66
HOG + Attention	20-fold	070	0.59	0.60	0.743 (± 0.050)	0.68
TopoBoW + XGBOOST	20-fold	0.88	0.87	0.87	0.882(± 0.051)	0.94
TopoBoW	20-fold	**0.97**	**0.98**	**0.97**	**0.975 (+/−0.033)**	**0.98**

## Data Availability

All datasets generated and analyzed during the previous study are available from the corresponding author on reasonable request.
